# Double-stranded DNA induces a prothrombotic phenotype in the vascular endothelium

**DOI:** 10.1038/s41598-017-01148-x

**Published:** 2017-04-25

**Authors:** Erik Gaitzsch, Thomas Czermak, Andrea Ribeiro, Yvonn Heun, Monica Bohmer, Monika Merkle, Hanna Mannell, Christian Schulz, Markus Wörnle, Joachim Pircher

**Affiliations:** 1 Medizinische Klinik und Poliklinik IV, Klinikum der LMU München, Munich, Germany; 2 Medizinische Klinik und Poliklinik III, Klinikum der LMU München, Munich, Germany; 3 Medizinische Klinik und Poliklinik I, Klinikum der LMU München, Munich, Germany; 4 Walter-Brendel-Center for Experimental Medicine, Klinikum der LMU München, Munich, Germany; 5 DZHK (German Center for Cardiovascular, Research) partner site Munich Heart Alliance, Munich, Germany

## Abstract

Double-stranded DNA (dsDNA) constitutes a potent activator of innate immunity, given its ability to bind intracellular pattern recognition receptors during viral infections or sterile tissue damage. While effects of dsDNA in immune cells have been extensively studied, dsDNA signalling and its pathophysiological implications in non-immune cells, such as the vascular endothelium, remain poorly understood. The aim of this study was to characterize prothrombotic effects of dsDNA in vascular endothelial cells. Transfection of cultured human endothelial cells with the synthetic dsDNA poly(dA:dT) induced upregulation of the prothrombotic molecules tissue factor and PAI-1, resulting in accelerated blood clotting *in vitro*, which was partly dependent on RIG-I signalling. Prothrombotic effects were also observed upon transfection of endothelial cells with hepatitis B virus DNA-containing immunoprecipitates as well human genomic DNA. In addition, dsDNA led to surface expression of von Willebrand factor resulting in increased platelet-endothelium-interactions under flow. Eventually, intrascrotal injection of dsDNA resulted in accelerated thrombus formation upon light/dye-induced endothelial injury in mouse cremaster arterioles and venules *in vivo*. In conclusion, we show that viral or endogenous dsDNA induces a prothrombotic phenotype in the vascular endothelium. These findings represent a novel link between pathogen- and danger-associated patterns within innate immunity and thrombosis.

## Introduction

The innate immune system constitutes a key response to both invading pathogens and sterile injury by recognition of pathogen associated- or danger associated molecular patterns (PAMPs or DAMPs, respectively). In this context lipopolysaccharides (LPS), peptidoglycans, high-mobility group protein-1 (HMGB1), double stranded DNA (dsDNA) and others are released into the circulation^1–3^. dsDNA is a powerful activator of the innate immune system^4^ and acts via several so called pattern-recognition receptors such as TLR-9 (toll-like receptor 9), AIM2 (absent in melanoma 2), DAI (DNA-dependent activator of IRFs), RIG-I (after transformation of DNA by RNA polymerase III)^4, 5^, and most recently Interferon-γ-inducible protein16 (IFI16) and cGAMP synthase (cGAS) have been discovered and shown to recognize intracellular dsDNA^6^. While the dsDNA-mediated immune response has been extensively studied in immune cells, little is known so far about the pathophysiological relevance of dsDNA for the vascular endothelium.

dsDNA plays a central role in viral infections such as hepatitis B, which often manifest as systemic diseases involving several organs including the vascular system. Morbidity and mortality result in part from vasculitis^7, 8^ but also from thrombotic complications including fatal thrombo-embolic events, such as myocardial infarction and ischemic stroke^9, 10^. Moreover viral infections are likely to play a role in the pathophysiology of atherosclerosis^11^. Even though not primarily considered part of the immune system, the endothelium as the inner layer of blood vessels plays a major role in host defense constituting an anatomical and functional barrier for pathogens to invade tissues. Additionally, the endothelium has essential function in suppressing inflammation and thrombosis by controlling vascular tone and function^12^. Endothelial inflammation leads to disruption of the haemostatic balance towards a prothrombotic state with increased risk of thrombo-embolic events^13^. Activated ECs are known to facilitate blood coagulation by down-regulation of antithrombotic proteins, such as thrombomodulin, tissue plasminogen activator or endothelial protein C receptor and by increased expression of procoagulant proteins such as the antifibrinolytic plasminogen activator inhibitor-1 (PAI-1) or tissue factor (TF)^14^.

We and others have previously shown, that the vascular endothelium is able to sense intracellular dsDNA and can exert a strong inflammatory response^15, 16^. In this study we investigated prothrombotic effects of dsDNA in the vascular endothelium.

## Results

### Double-stranded DNA led to nuclear translocation of transcription factors IRF3 and NF-κB

**Figure 1:**
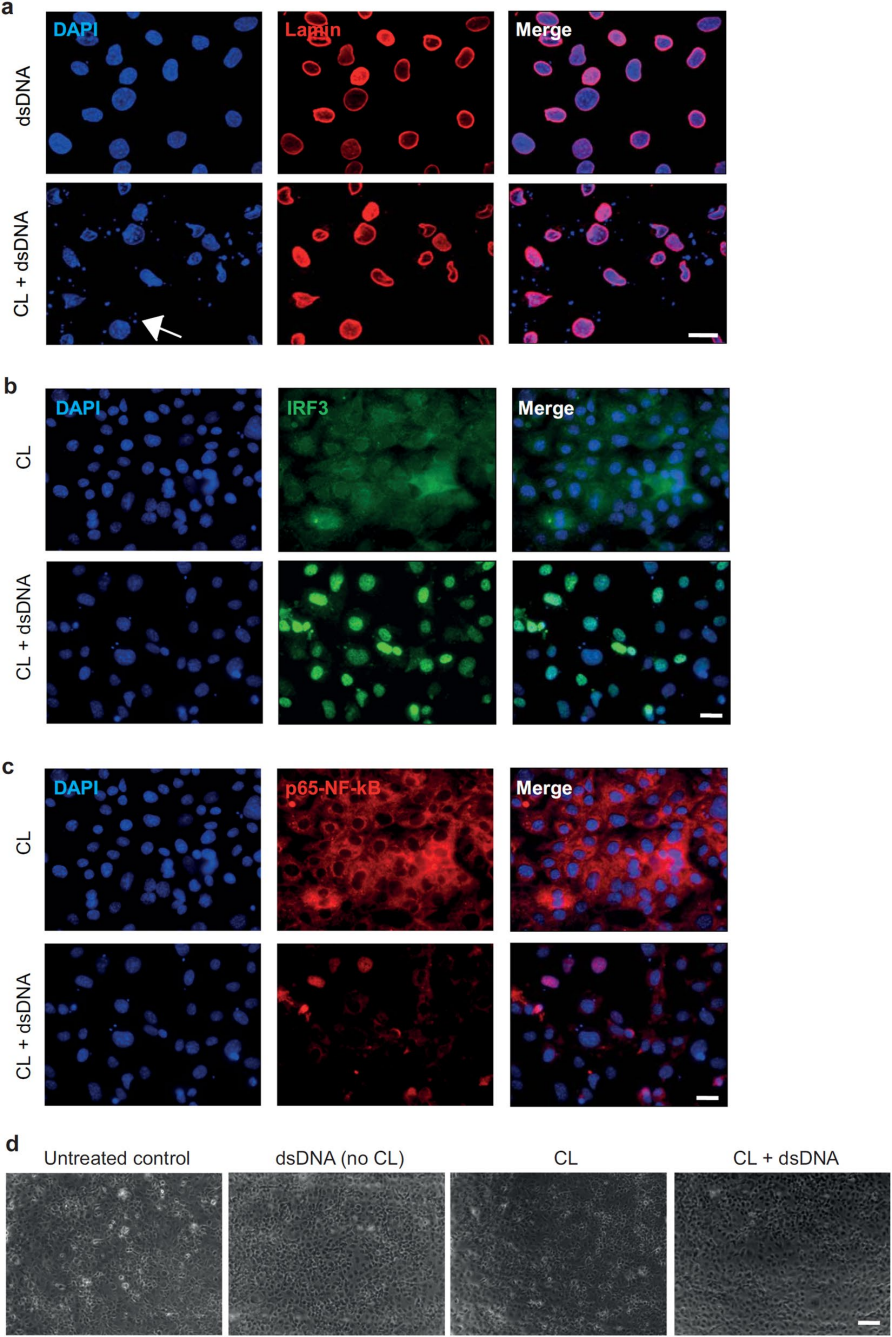
Double-stranded DNA led to nuclear translocation of transcription factors IRF3 and NF-κB. (**a**) Immunofluorescent staining of cultured endothelial cells with DAPI (blue) and anti-Lamin-antibody (red) showed transfection of poly(dA:dT) into the intracellular compartment after complex formation with cationic lipids. Scale bar is 10 µm. (**b**,**c**) Transfection of endothelial cells with poly(dA:dT) led to nuclear translocation of transcription factors IRF3 (**b**) and to a lesser extent of NF-κB (**c**). Cells were stained with antibodies against IRF3 (green) and p65-subunit of NF-κB (red). Scale bar is 10 µm. (**d**) Representative bright field images upon treatment of endothelial cells with poly(dA:dT) with or without cationic lipids. Scale bar is 100 µm.

Human microvascular endothelial cells (HMEC) were treated with poly(dA:dT) 5 µg/mL with or without complexation with cationic lipids (Lipofectamine 2000) for 6 hours and stained with DAPI and anti-Lamin-antibody. Only poly(dA:dT) complexed with cationic lipids but not poly(dA:dT) alone led to uptake of dsDNA into the intracellular compartments (representative images in Fig. 1a). Transfection of endothelial cells with poly(dA:dT) led to nuclear translocation of transcription factors IRF3 and to a lesser extent of NF-κB as shown by immunofluorescent staining (representative images in Fig. 1b and c). To check integrity of the endothelial cell monolayer 8 hours after transfection with poly(dA:dT) bright field images were taken, which showed comparable intact monolayers in cells treated with poly(dA:dT) with or without cationic lipids (representative images in Fig. 1d).

### Double-stranded DNA induces expression of prothrombotic genes in vascular endothelial cells

**Figure 2:**
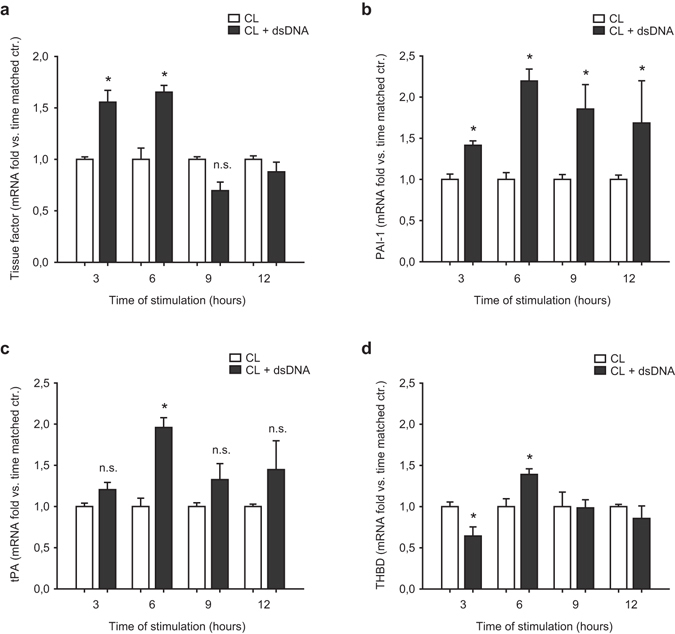
Double-stranded DNA induces expression of prothrombotic genes in vascular endothelial cells. (**a**,**b**) Expression of the prothrombotic molecules tissue factor (**a**) and Plasminogen activator inhibitor-1 (PAI-1, **b**) as assessed by RT-PCR upon transfection of vascular endothelial cells with poly(dA:dT). (**c**,**d**) Expression of the antithrombotic molecules tissue plasminogen activator (tPA, (**c**)) and thrombomodulin (THBD, (**d**)) upon transfection of vascular endothelial cells with poly(dA:dT); (*P < 0.05 vs. respective time-matched control, n = 4).

Next, we measured expression of pro- and antithrombotic genes by real-time PCR. Transfection of human microvascular endothelial cells (HMEC) with poly(dA:dT) (5 µg/mL) induced time-dependent expression of tissue factor as well as plasminogen activator inhibitor-1 (PAI-1) with a maximal relative increase at 6 or 12 hours, respectively (Fig. 2a and b). We also observe significantly increased expression of the fibrinolytic molecule tissue plasminogen activator (tPA) after 6 hours of cell transfection with poly(dA:dT) (Fig. 2c), while thrombomodulin (THBD) expression was slightly increased after 6 hours of transfection with poly(dA:dT) after an initial decrease after 3 hours (Fig. 2d).

### Double stranded DNA induced prothrombotic proteins and accelerates endothelial dependent blood clotting *in vitro*

**Figure 3:**
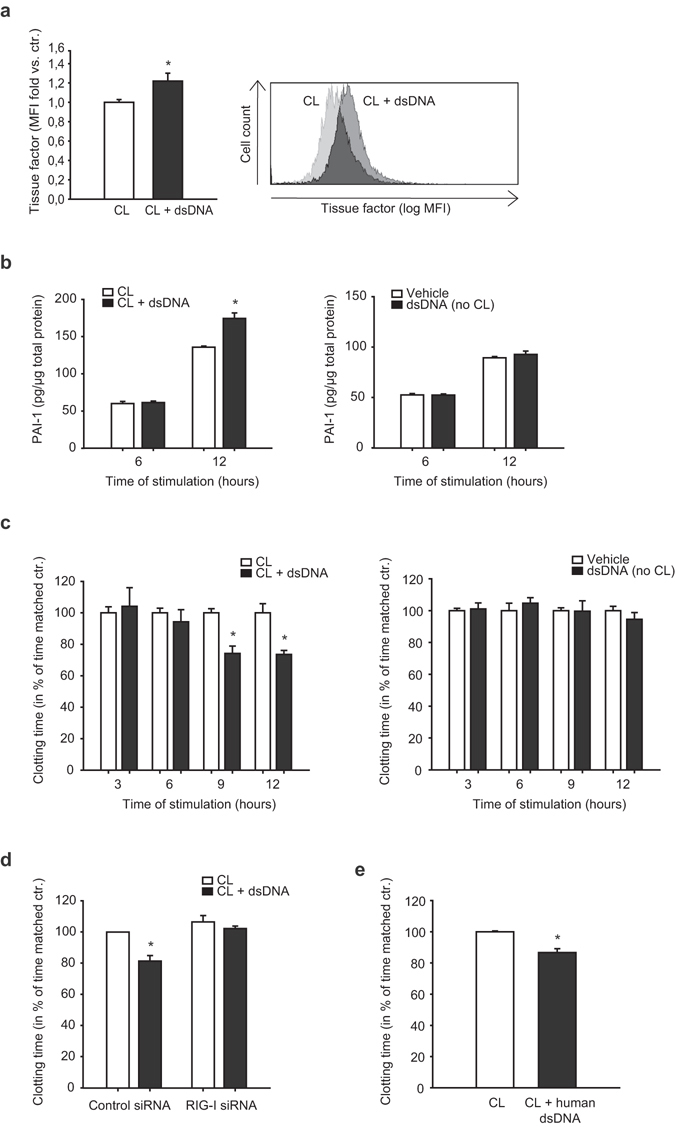
Double stranded DNA induces prothrombotic proteins and accelerates endothelial dependent blood clotting *in vitro*. (**a**) Tissue factor surface expression was assessed by flow cytometry 12 hours after transfection of endothelial cells with poly(dA:dT) (*P < 0.05 vs. control, n = 8, MFI mean fluorescence intensity). Representative histogram is shown on the right. (**b**) PAI-1 release by endothelial cells was measured by ELISA 6 and 12 hours after transfection with poly(dA:dT), PAI-1 release after stimulation of endothelial cells with poly(dA:dT) alone (i.e. without cationic lipids) is shown on the right (*P < 0.05 vs. time matched control, n = 5). (**c**) Stimulation of whole blood with endothelial cell lysates transfected with poly(dA:dT) accelerated blood clotting time compared to stimulation with untreated cell lysates as measured by thromboelastometry. Stimulation of whole blood with lysates of endothelial cells treated with poly(dA:dT) alone (i.e. without cationic lipids) had no effect on blood clotting time (right) (*P < 0.05 vs. time matched control, n = 4). (**d**) The prothrombotic effect of poly(dA:dT) after 12 hours was partly reversed by siRNA-silencing of RIG-I (*P < 0.05 vs. time matched control, n = 4). (**e**) A similar prothrombotic effect was observed 12 hours after transfection of endothelial cells with human genomic DNA (*P < 0.05 vs. time-matched control, n = 4).

Next, upregulation of prothrombotic molecules tissue factor and PAI-1 was assessed on protein level. Tissue factor surface expression on the cell membrane was significantly increased after stimulation with poly(dA:dT) for 12 hours as assessed by flow cytometry (Fig. 3a). PAI-1 release by endothelial cells as measured by ELISA was significantly increased 12 hours after transfection with poly(dA:dT) but not after 6 hours as compared to respective time-matched controls. In contrast, PAI-1 release was not influenced after stimulation of endothelial cells with poly(dA:dT) alone, i.e. without cationic lipids (Fig. 3b).

In order to functionally analyze prothrombotic properties of endothelial cells, an endothelial dependent blood clotting assay was performed. Lysates of poly(dA:dT) transfected endothelial cells accelerated blood clotting time compared to time-matched control cells (Fig. 3c, left). Stimulation of whole blood with lysates of endothelial cells treated with poly(dA:dT) alone (i.e. without cationic lipids) had no effect on blood clotting time (Fig. 3c, right). Similar to cells transfected with the synthetic dsDNA analogue poly(dA:dT), lysates of endothelial cells transfected with human genomic DNA from peripheral human leukocytes also induced a significantly accelerated blood clotting compared to control cells after 12 hours (Fig. 3e). The prothrombotic effect of poly(dA:dT) after 12 hours was partly reversed after prior transfection of endothelial cells with siRNA silencing RIG-I receptor (Fig. 3d).

### Double stranded DNA induces vWF upregulation and platelet tethering *in vitro*

**Figure 4:**
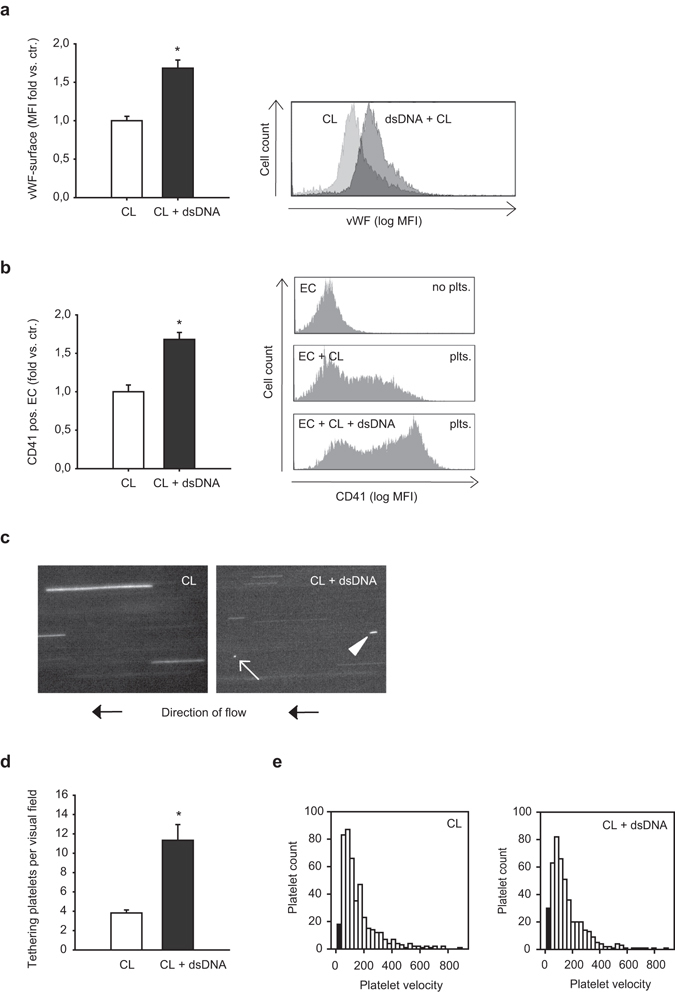
Double-stranded DNA induces vWF upregulation and platelet tethering *in vitro*. (**a**) Transfection of endothelial cells with poly(dA:dT) for 12 hours caused upregulation of von Willebrand factor on the endothelial cell surface (*P < 0.05 vs. control, n = 6; MFI mean fluorescence intensity). Representative histogram is shown on the right. (**b**) Co-cultivation of endothelial cells transfected with poly(dA:dT) for 12 hours and non-stimulated isolated platelets resulted in increased adhesion of platelets to endothelial cells, as assessed by FACS analysis (*P < 0.05, vs. control, n = 6). Representative histograms are shown on the right. (**c**) Representative snap shots of flow chamber assays with transfusion of fluorescently labeled platelets simulating vascular shear stress of 1 dyn/cm^2^. Platelet tethering (white arrow) and very slow rolling platelets (white arrowhead) were analyzed after stimulation of endothelial cells with poly(dA:dT) for 12 hours. (**d**) Quantitative analysis showed increased number of tethering platelets (*P < 0.05 vs. control, n = 9) as well as (**e**) increased number of very slow rolling platelets (as displayed by the black columns in the histograms showing platelet velocity profiles) upon transfection with poly(dA:dT).

Von Willebrand factor (vWF) surface expression and platelet adhesion were analyzed in primary human umbilical vein endothelial cells (HUVEC). Transfection of endothelial cells with poly(dA:dT) significantly increased surface expression of vWF after 12 hours as assessed by flow cytometry (Fig. 4a). To investigate the functional relevance of this observation, interactions between endothelial cells and platelets were examined in a model of static adhesion. Therefore endothelial cells pre-treated with poly(dA:dT) for 12 hours were then co-cultivated with freshly isolated platelets from healthy volunteers for 6 hours. Endothelial cells transfected with dsDNA showed significantly increased numbers of adherent platelets as compared to non-stimulated cells (Fig. 4b). In order to confirm our findings in a more physiological setting, we established a flow based assay of platelet endothelium interaction. Therefore freshly isolated human platelets were labeled with Calcein and flushed over cultured endothelial cells in a flow chamber simulating a vascular shear stress of 1 dyn/cm^2^ and platelet-endothelial cell interactions were analyzed by immunofluorescence microscopy (Fig. 4c). Poly(dA:dT) transfected endothelial cells showed significantly increased amount of tethering platelets compared to non stimulated cells (Fig. 4d). Additionally the number of very slow rolling platelets was higher on poly(dA:dT) transfected endothelial cells, however (considering the high number of overall transfused platelets) the median velocity of platelets was not different in both groups (Fig. 4e).

### Double stranded DNA accelerates microvascular thrombosis *in vivo*

**Figure 5:**
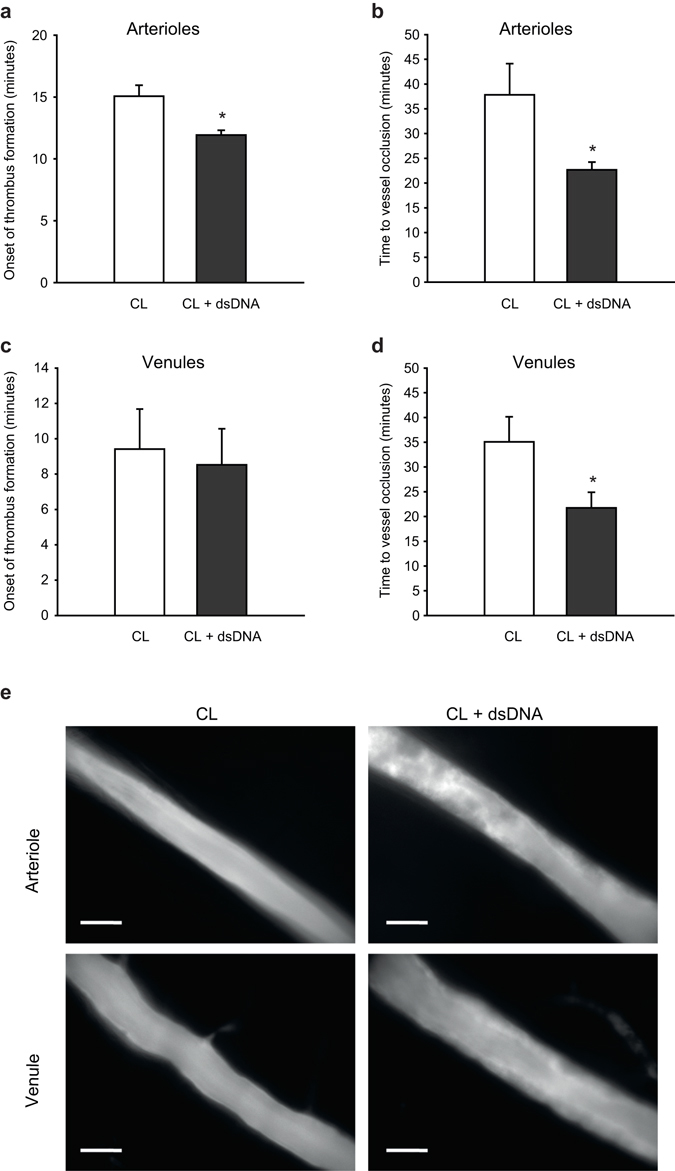
Double-stranded DNA accelerates microvascular thrombosis *in vivo*. Thrombus formation *in vivo* was investigated by phototoxic (light/dye-induced) vessel injury of microvessels in the mouse cremaster muscle. Intrascrotal injection of poly(dA:dT) (5 µg DNA 12 hours prior to the experiment) resulted in a more rapid onset of thrombus formation in arterioles (**a**) but not in venules (**c**). Time to complete vessel occlusion was significantly accelerated after poly(dA:dT) injection in both arterioles (**b**) and venules (**d**). (**e**) Representative images show thrombus formation in mouse cremaster arterioles (20 min after start of injury) and mouse cremaster venules (10 min after start of injury) with markedly increased thrombus formation in poly(dA:dT) treated animals (right pictures) as compared to control animals (left pictures). Scale bar is 50 µm. (*P < 0.05 vs. control, n = 6 animals each).

To investigate the effects of double stranded DNA on thrombus formation *in vivo*, 10 μg poly(dA:dT) complexed with 10 μl of Lipofectamine or transfection reagent alone (control group) was injected into the scrotum of C57Bl/6 mice. Intravital microscopy of cremaster muscle vessels was performed 12 hours after injection and thrombus formation was induced by light/dye-injury after injection of FITC-labeled dextran. Vessel diameters did not differ significantly between both groups and were 53 +/− 5 µm vs. 48 +/− 6 µm in venules and 51 +/− 9 µm vs. 50 +/− 3 µm in arterioles control treated vs. dsDNA treated animals respectively. dsDNA stimulation significantly accelerated thrombus formation *in vivo* resulting in a reduced time until onset of thrombus formation after injury in arterioles (Fig. 5c) and eventually also in significantly reduced time until complete thrombotic vessel occlusion with flow cessation in both venules and arterioles (Fig. 5a and b, representative images in Fig. 5e).

### Hepatitis B virus DNA-containing immunoprecipitates induce a prothrombotic phenotype

**Figure 6:**
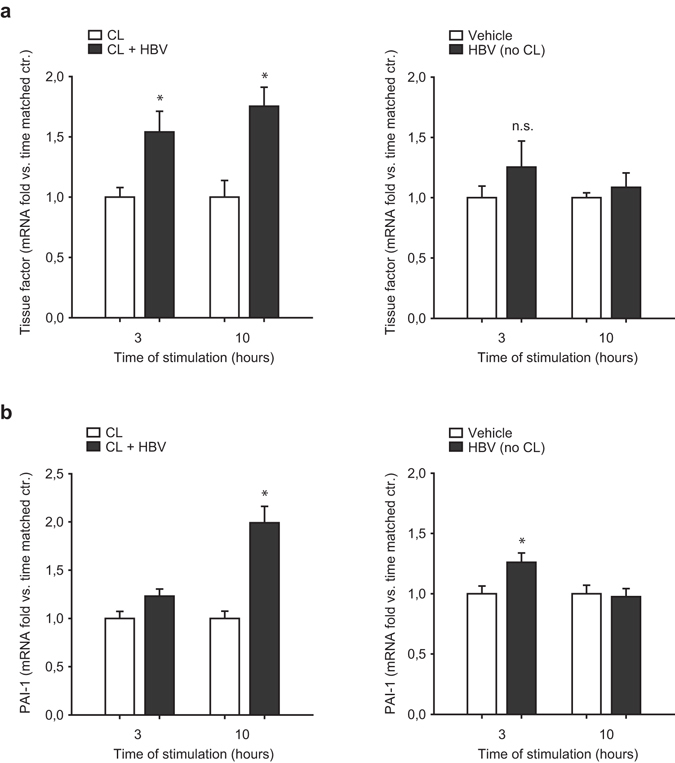
Hepatitis B virus DNA-containing immunoprecipitates induce a prothrombotic phenotype. Endothelial cells were transfected with HBV-DNA containing immunoprecipitates isolated from a patient with ongoing hepatitis B infection and associated polyarteritis nodosa with a high viral load. HBV-DNA containing immunoprecipitates resulted in upregulation of tissue factor starting 3 hours after transfection (**a**) as well as PAI-I at 10 hours after transfection (**b**) as analyzed by RT-PCR. Expression of tissue factor and PAI-1 after stimulation of endothelial cells with HBV-DNA alone (i.e. without cationic lipids) is shown on the right ((**a** and **b**), respectively). (*P < 0.05 vs. control).

To translate our findings into a clinical context we investigated whether Hepatitis B virus DNA induces prothrombotic effects in the vascular endothelium. Therefore we transfected microvascular endothelial cells with hepatitis B virus (HBV)-containing immunoprecipitates, that were collected during plasmapheresis from a patient with HBV-associated polyarteritis nodosa. Similar to transfection with the synthetic analogue poly(dA:dT) HBV-containing immunoprecipitates exerted a prothrombotic phenotype in transfected endothelial cells resulting in upregulation of tissue factor already after 3 hours and upregulation of PAI-1 expression after 10 h as assessed by real-time PCR (Fig. 5a and b respectively, left images). HBV-DNA alone (i.e. without cationic lipids) had no effect on expression of tissue factor and PAI-1 expression compared to time-matched controls (Fig. 6a and b, respectively, right images).

## Discussion

In this study, we show direct prothrombotic effects of intracellular double stranded DNA (dsDNA) in the vascular endothelium. dsDNA led to upregulation of the procoagulatory proteins tissue factor and PAI-1 and increased surface expression of vWF and eventually resulted in accelerated blood clotting *in vitro* and thrombus formation in a model of endothelial injury *in vivo*. Similar effects were observed after transfection of endothelial cells with hepatitis B virus DNA containing immunoprecipitates and with endogenous human DNA.

In previous work we showed that dsDNA, both from viral origin as well as endogenous DNA, can induce pro-inflammatory effects in endothelial cells resulting in upregulation of inflammatory cytokines such as IL-6, IL-8, MCP-1, RANTES, IP-10 and IFN-β, as well as the adhesion molecules ICAM-1 and VCAM-1 on human endothelial cells^15^. Furthermore, dsDNA has been described to induce TNFα release from endothelial cells and thereby promoting leukocyte adhesion^16^. Similar effects have also been observed in glomerular endothelial cells where dsDNA functionally increased albumin permeability^17^.

In this study we show intracellular dsDNA leads to upregulation of tissue factor, a crucial protein in the activation of the extrinsic pathway of the coagulation cascade^18^. Tissue factor initiates the extrinsic pathway of the coagulation cascade and contributes to thrombus growth and stabilization^19^. Additionally, under certain pathophysiological conditions such as sickle cell disease or antiphospholipid syndrome tissue factor-positive endothelial microparticles have been observed, that can be recruited to the sites of vascular injury and contribute to increased thrombin generation^20, 21^. Physiologically resting endothelial cells express, if at all, very little amounts of tissue factor, however, the protein is upregulated under inflammatory conditions^14, 22–24^. Indeed, on a functional level we observed, that stimulation of endothelial cells with dsDNA resulted in accelerated blood clot formation in an endothelial-dependent *in vitro* whole blood clotting assay, which has been shown to be, at least in part, tissue factor dependent^25^.

dsDNA furthermore induced expression of plasminogen activator inhibitor-1 (PAI-1), which is the major physiologic inhibitor of tissue-type plasminogen activator in plasma and thereby serves as an endogenous mechanism to prevent intravasal thrombosis^26^. PAI-1 levels are elevated in many diseases associated with increased risk of ischemic cardiovascular events^27, 28^ and have recently been shown to be relevant cardiovascular events in chronic hepatitis C infections^29^.

Interestingly, we also observed changes in antithrombotic molecules. While thrombomodulin expression seems to be less influenced by intracellular dsDNA, we observe an upregulation of the fibrinolytic molecule tPA at certain time points, indicating compensatory mechanisms. However, in our functional experiments dsDNA shows an overall prothrombotic effect, which could be explained by a relative stronger expression of the prothrombotic or antifibrinolytic molecules such as PAI-1, a strong prothrombotic counterpart to tPA^14^.

Several molecular mechanisms seem to be involved in dsDNA signaling in endothelial cells. Consistent with findings of previous studies in similar cells we found intracellular dsDNA to induce nuclear translocation of the transcription factor IRF3 and to lesser extent also NF-κB^16^. These transcription factors can upregulate proinflammatory and prothrombotic genes. The list of possible receptors being involved in the intracellular sensing of dsDNA is long and further expanding^4–6^. Several of these receptors including AIM2, DAI, TLR9 or RIG-I are expressed in endothelial cells and intracellular dsDNA signaling is likely dependent on more than a single receptor and pathway^30^. In a previous study by our group we could see that upregulation of some inflammatory markers is dependent on TLR9 signaling^15^. Considering the slighter prothrombotic effect of dsDNA after transfection of endothelial cells with siRNA and dsDNA, it was partly reversed with siRNA silencing RIG-I receptor, indicating this receptor to be relevant for prothrombotic effects.

Besides pro-coagulatory effects, dsDNA in endothelial cells induced surface expression of von-Willebrand-factor (vWF) which can bind GPIb-receptor on platelets resulting in platelet-endothelium interactions. Indeed, platelet-endothelial cell-aggregates were significantly increased after prior stimulation of endothelial cells with dsDNA. Moreover, transfection of primary human endothelial cells cultivated in flow chambers led to increased platelet tethering under dynamic conditions. Platelet-endothelial interactions via vWF-GPIb or CD40-CD40L, even when remaining transient, have been shown to further activate the endothelium and thereby increase inflammatory effects resulting in thrombosis but also acceleration in development of atherosclerotic lesions^31, 32^.

Eventually, dsDNA stimulation led to increased thrombus formation upon light/dye-induced endothelial injury in mouse cremaster vessels *in vivo*. In this model the endothelium is not immediately disrupted, such as in the case of ferric chloride based injury models, but gradually exposed to increasing oxidative stress. Thus, *in vitro* observed effects of dsDNA are likely to contribute to the prothrombotic effects. However, additional effects including expression of other endothelial cell surface molecules, such as inflammatory adhesion molecules (e.g. ICAM-1, VCAM-1) or P-Selectin, but also redistribution or interplay between surface molecules may foster prothrombotic effects. Furthermore, other mechanisms such as modulation of endothelial miRNA expression, e.g. miR-126 might influence endothelial injury and consecutive vascular thrombosis^33^. While accelerated complete thrombotic vessel occlusion was consistent in arterioles and venules, the onset of thrombus formation was accelerated only in arterioles. Among the differences between mechanisms contributing to thrombosis in arterioles and venules lower shear rates as well as a considerable role of leukocytes in venules are the most important ones. vWF is particularly important for platelet adhesion at higher shear rates, which could explain significant differences in the onset of thrombus formation in arterioles but not in venules. Notably we observed increased surface expression of vWF *in vitro* upon stimulation of dsDNA which would support this hypothesis.

Poly (dA:dT) when transfected into cells mimics effects of viral DNA and has been used as an analog for viral DNA in previous studies by ourselves and others^15–17^. Viral infections, such as hepatitis B are often associated with vasculitis and subsequently thromboembolic complications^7, 8, 34^. We therefore used immunoprecipitates isolated from a patient with ongoing hepatitis B infection and associated systemic vasculitis, to stimulate primary endothelial cells. Indeed, with regard to slight differences in the kinetics, we do see a similar upregulation of prothrombotic genes as compared with synthetic dsDNA stimulation, suggesting a putative role of dsDNA triggered endothelial activation in viral infections. Our observations could help to elucidate the so far incompletely understood relation between viral infections and atherothrombotic diseases.

Apart from viral infections, dsDNA can be released into the bloodstream from damaged host cells in the context autoimmune diseases, tumor lysis or formation of neutrophil extracellular traps (NETs). We have previously shown that human genomic dsDNA can enter the intracellular compartment of endothelial cells under certain circumstances and induce an inflammatory response^15^. In this study we demonstrate that transfection of cultured endothelial cells with human genomic dsDNA accelerates blood clotting *in vitro*. The induced phenotype is therefore similar to the one observed after poly(dA:dT) treatment, however the extent of the prothrombotic effect observed was less pronounced as compared to stimulation with poly(dA:dT). This prothrombotic phenotype might thereby contribute to thrombo-embolic complications in inflammatory autoimmune disorders that are well known to be associated with an increased risk of atherothrombotic events^35–37^.

In conclusion we could show for the first time a direct pathophysiological role of viral as well as genomic intracellular dsDNA in thrombosis and haemostasis by endothelial mediated mechanisms. Our results are in line with the growing evidence, that non-primary immune cells, such as endothelial cells, play an important role in the recognition and reaction to pathogen- and danger associated molecular patterns within the innate immune system. Ultimately, our findings represent a novel link in the increasingly recognized reciprocal connection between innate immunity and thrombotic disorders and therefore could be relevant for therapeutic decisions in patients with inflammatory and cardiovascular diseases.

## Materials and Methods

### Chemicals and Antibodies

Poly(dA:dT) was from Invivogen (Toulouse, France), human genomic DNA was from AMS Biotechnology (Milton Park, UK), Lipofectamine 2000 transfection agent was from Invitrogen (Carlsbad, USA), Accutase was from PAA (Cölbe, Germany), p65-NF-kB antibody and rabbit IRF3 antibody were from (Cell Signaling Technology, USA), goat lamin A/C antibody was from Santa Cruz Biotechnology (USA). DAPI, Alexa Fluor 488 Chicken Anti-Rabbit IgG, Alexa Fluor 546 Donkey Anti-goat IgG were all from Invitrogen (UK). Predesigned TaqMan primers for tissue factor, PAI-1, tPA, thrombomodulin and GAPDH were from Applied Biosystems (Carlsbad, California, USA), PAI-1 ELISA-Kit was from Abcam (UK), RIG-I-siRNA and negative control siRNA was from Ambion/ThermoFisher (USA). Calcein-AM was from Merck-Millipore (Darmstadt, Germany), anti-human tissue factor-FITC- and anti-human vWF-FITC-antibodies and respective isotype-controls were from AbD Serotec (Oxford, UK), anti-human CD41-APC-antibody was from BD (USA). All other chemicals unless otherwise indicated in the method section were from Sigma Aldrich (Taufkirchen, Germany).

### Isolation of hepatitis B virus-DNA (HBV-DNA) containing immunoprecipitates

HBV-DNA containing immunoprecipitates were isolated from a patient with a hepatitis B associated polyarteritis nodosa with a high viral load, during routine plasmapheresis treatment as described previously^15^. The concentration of HBV-DNA used for stimulation was 2.1 × 10^6^ geq/mL as confirmed by real-time-PCR. Written informed consent for the collection of plasma samples was obtained and the procedure was approved by the university ethics review board.

### Cell culture and stimulation of endothelial cells

Human microvascular endothelial cells (HMEC) were provided by Ades *et al*.^38^, human umbilical vein endothelial cells (HUVECs) were isolated and cultivated as described previously^23^. Briefly, cells were cultured in M199 media supplemented with 10% fetal calf serum, 10% endothelial growth media (PromoCell, Heidelberg, Germany), and 1% penicillin/streptomycin. The procedure was approved by the university ethics review board. dsDNA stimulation was performed as previously described [5]. Briefly, 5 µg/mL poly(dA:dT), or for indicated experiments 5 µg/mL human genomic DNA or HBV-DNA containing immunoprecipitates were complexed with 1 µl of Lipofectamine 2000 in order to achieve intracellular transfection with dsDNA. For stimulation with HBV-containing immunoprecipitates, culture dishes with endothelial cells were additionally centrifuged at 1000 g for 45 min after addition of the immunoprecipitates in order to increase efficiency of viral infection. siRNA knockdown of RIG-I was performed as described previously^15^.

### Immunofluorescence microscopy

For immunofluorescence experiments, HMEC were grown to confluence in 8-well microscope µ-slides (Ibidi, Germany) and stained as previously described^39^. After treatment of cells as indicated cells were washed with PBS followed by fixation and permeabilization in 100% methanol or for 30 min. Fixed cells were washed again with PBS and blocked with 5% BSA in PBS for 1 h at room temperature. Cells were then incubated with first antibodies diluted 1:200 in blocking solution (goat lamin A/C antibody, mouse p65-NF-kB antibody, rabbit IRF3 antibody) for one hour at room temperature. Afterwards, cells were washed three times with PBS and subsequently incubated with the secondary antibody diluted 1:400 in blocking solution (Alexa Fluor 488 Chicken Anti-Rabbit IgG and Alexa Fluor 546 Donkey Anti-goat IgG) and with DAPI for 30 min. Cells were washed and kept in PBS for microscopy. Images were taken with an Axiovert 200 M microscope with ApoTome (Zeiss, Jena, Germany).

### Real-time PCR

RNA isolation from endothelial cells and real-time polymerase chain reaction (PCR) were performed as described previously^23^. Commercially available pre-developed TaqMan reagents were used for the human target genes PAI-1, Tissue factor, tPA and thrombomodulin. GAPDH was used as reference housekeeping gene.

### Flow cytometry

Tissue factor and von Willebrand factor were measured on endothelial cell surface by flow cytometry. Endothelial cells after indicated stimulation were washed and detached from dishes using accutase and stained with fluorescent antibodies or respective isotype controls for 30 minutes at 37 °C. Analysis was performed using a FACS Canto II flow cytometer (BD, USA).

### ELISA

PAI-1 protein levels were measured in supernatants of cultured endothelial cells after indicated treatments by ELISA according to the manufacturer’s protocol.

### Blood donors

All blood donors were healthy volunteers, who had given written consent and had not taken any medications for at least 10 days. The investigation was carried out according to the principles of the Helsinki-Declaration.

### Endothelial-dependent blood clotting assay

Endothelial cells were stimulated as indicated and then lysed with 15 mM n-octyl-D-glycopyranosidase in 0.1 M imidazol buffer; 20 μL of cell lysate and 20 μL of 200 mmol/L CaCl2 for re-calcification were added to 300 μL of citrated (3.13% sodium citrate) human whole blood from healthy volunteers, and clotting time was measured by thromboelastometry (ROTEG; Tem Innovations, Munich, Germany).

### Platelet preparation

Platelet isolation was performed as previously described^40^. Platelet rich plasma was obtained by centrifugation of anticoagulated (3.13% sodium citrate) whole blood at 340 *g* for 15 minutes. After another centrifugation step at 600 *g* for 10 minutes in the presence of 2 ng/mL Prostaglandin, platelets were washed and resuspended in calcium-free modified Tyrode buffer (138 mmol/L NaCl, 2.7 mmol/L KCl, 12 mmol/L NaHCO3, 400 mmol/L Na2HPO4, 1 mmol/L MgCl2, 5 mmol/L *D*-glucose, and 5 mmol/L HEPES) and adjusted to the concentration required for the respective experiment. Platelet counts were obtained using a resistance particle counter (Coulter Z2, Beckman Coulter, Krefeld, Germany).

### Platelet-endothelial cell-aggregates

Endothelial were cultivated and stimulated as indicated. After removing the transfection medium and several washing steps with PBS isolated washed platelets from healthy donors (prepared as described above) were co-cultivated with endothelial cells for 6 hours. Cells were then washed with PBS in order to remove non-adherent platelets and detached with accutase. Platelet-endothelial cell-aggregates were measured by staining with anti-human CD41-APC antibody and quantified by flow cytometry.

### Flow chamber assay

Endothelial cells were cultivated and stimulated as indicated in a µ-slide from IBIDI (Martinsried, Germany). Freshly isolated human platelets were labeled with Calcein (10 µM for 30 min) and perfused over the cultured endothelial cells at a shear rate of 1 dyn/cm^2^. Live immune-fluorescence imaging was performed using a Zeiss Axiotech Vario microscope (Carl Zeiss, Oberkochen, Germany). Images were recorded with a digital camera (AxioCam HSm; Carl Zeiss). From the resulting length of the platelet trace in single images, velocities of single platelets were calculated by using the exposure time of each single picture. Platelet-endothelial cell-interaction was expressed by analysis of the amount of tethering platelets (platelets not moving in at least one single picture) as well as by analysis of frequency histograms consisting of all platelet velocities.

### Animals

Animal experiments were performed in wildtype C57Bl/6 mice, which were purchased from Charles River (Sulzfeld, Germany). Surgical procedures were performed under short-term anesthesia induced by a single intraperitoneal injection of Midazolam 5 mg/kg (Ratiopharm, Germany), Fentanyl 0.05 mg/kg (CuraMED Pharma, Germany), and Medetomidinehydrochloride 0.5 mg/kg (Pfizer, Germany; produced by Orion Pharma, Finland) diluted in 0.9% NaCl. All experiments were conducted in accordance with the German animal protection law and approved by the district government of Upper Bavaria. The investigation conforms to the Directive 2010/63/EU of the European Parliament.

### Intravital assessment of microvascular thrombosis

Microvascular thrombosis *in vivo* was investigated in arterioles and venules of the mouse cremaster muscle using a light/dye-injury model as previously described^41, 42^. After anesthesia all surgical procedures were conducted on a thermo-controlled plexi-glass stage to maintain body temperature at 37 °C with cover slips for microscopy. Intravital fluorescence microscopy was performed using a modified microscope (Zeiss Axiotech Vario, Carl Zeiss Microscopy GmbH, Germany). Images were recorded with a digital camera (AxioCam HSm, Carl Zeiss Germany) and analyzed with “AxioVision” (Carl Zeiss Microscopy GmbH, Germany). After the surgical preparation of the cremaster muscle 1–2 arterioles and 1–2 venules per cremaster with a diameter of around 50 μm were chosen and FITC-dextran (average molecular weight 150,000 (Sigma-Aldrich, Germany)) in a concentration of 5% in PBS was given in a dose of 1 μl/g mouse via a tail vein catheter. Light with a wave length of 450–490 nm was used to excite the fluorescent dye inducing photochemical injury of the endothelial cell layer. Two end points were defined, first the onset of thrombus formation and second the time until total cessation of blood flow (occlusion time).

### Statistical Analysis

Data were analyzed using Student *t*-test to compare normally distributed variables and Mann–Whitney *U* test when normal distribution was not given. All data are expressed as mean ± SEM. Differences were considered significant when the error probability was *P* < 0.05.
